# An update on the characterization of immunoglobulin loci in *Ambystoma mexicanum*

**DOI:** 10.3389/fimmu.2026.1736245

**Published:** 2026-02-23

**Authors:** Stephanie Saint Remy-Hernández, Diana Laura Pacheco-Olvera, Elizabeth Ernestina Godoy-Lozano, Juan Miguel-Ruiz, Juan Téllez-Sosa, Humberto Valdovinos-Torres, Nancy Rivas, Constantino López-Macías, Jesús Martínez-Barnetche

**Affiliations:** 1 Departamento de Parasitología, Posgrado en Ciencias Quimicobiológicas, Escuela Nacional de Ciencias Biológicas, Instituto Politécnico Nacional, México City, Mexico; 2 Unidad de Investigación Médica en Inmunoquímica, UMAE Hospital de Especialidades, Centro Médico Nacional Siglo XXI, Instituto Mexicano del Seguro Social, México City, Mexico; 3 Departamento de Bioquímica, Posgrado en Ciencias Quimicobiológicas, Escuela Nacional de Ciencias Biológicas, Instituto Politécnico Nacional, México City, Mexico; 4 Centro de Investigación sobre Enfermedades Infecciosas, Instituto Nacional de Salud Pública, Cuernavaca, Morelos, Mexico; 5 Departamento de Ciencias Computacionales, Tecnológico Nacional de México/CENIDET, Cuernavaca, Morelos, Mexico

**Keywords:** Ambystoma, comparative genomics, copy number variation, Ig loci, immunoglobulin genes

## Abstract

**Background:**

We previously reported the genomic characterization of immunoglobulin loci in *Ambystoma mexicanum* of the laboratory d/d white strain, where the IGH locus gene orientation was incompatible with VDJ recombination, suggesting scaffold orientation errors. A novel 29.1 Gbp *A. mexicanum* genome derived from an F1 cross between *A. mexicanum* and *A. tigrinum* (UKY_AmexF1_1) has recently been released, containing only 220 unmapped scaffolds. Here, we present an updated description of the immunoglobulin loci based on this improved genome assembly.

**Methods:**

Using our previously annotated AmbMex60DD sequences, we performed an alignment-based annotation followed by manual curation on the UKY_AmexF1_1 assembly. Gene models were further refined using RNA-Seq datasets from axolotl spleen, liver, lung, heart, and gill tissues.

**Results:**

The IGH locus was mapped at 350–364 Mbp on chromosome 13q. Synteny is preserved between both genome versions, but in UKY_AmexF1_1, all IGH genes share the same transcriptional orientation. A major difference was observed in the lambda locus, which in the UKY_Amex_F1_1 genome contains 13 IGLC–IGLJ clusters comprising 60 IGLJ genes, compared with only three clusters in the AmbMex60DD genome. No kappa locus was detected in either assembly.

**Conclusion:**

This study confirms our previous findings and provides an example of intraspecies structural variation in adaptive immune receptor loci. It underscores the importance of well-assembled genomes and establishes the current *A. mexicanum* reference as a valuable resource for investigating immune evolution and function in axolotls and other vertebrates.

## 1 Introduction

Adaptive antibody-dependent immunity is based on the generation of a highly diversified, clonally expressed antigen receptor repertoire on the cell surface of B lymphocytes. The B cell repertoire is generated through somatic recombination events in the V, D, and J segments of immunoglobulin loci. Despite the commonalities in the generation of the vertebrate adaptive immune response repertoire diversity, there is considerable evolutionary plasticity in the structure of immune response loci, antibody classes, and the number of functional segments involved. Recently, intra-species variation, including single-nucleotide (SNV) and copy number variation (CNV), shapes the germ line repertoire in acquired immunity receptor loci. (1).

Most of our knowledge of the amphibian adaptive immune system comes from *Xenopus*, a well-studied anuran species. *A. mexicanum*, a caudate amphibian, is a model for development, tissue regeneration, and its large genome (32 Gbp) presents a highly complex assembly challenge (2).

To expand the taxonomic spectrum of immunoglobulin evolution, we recently performed the genomic characterization of the immunoglobulin loci in *A. mexicanum* (3), based on the AmbMex60DD genome (white d/d laboratory strain). We reported unusual Ig loci configurations, such as the orientation of the IGHJ-IGHC cluster respective to the IGHV cluster that could be the result of misassembly, as well as a substantial number of unmapped V genes. A new axolotl genome sequence (UKY_AmexF1_1) derived from an *A. mexicanum* x *A.tigrinum* F1 individual was released in 2024. In the latest version, only 220 scaffolds are unmapped to chromosomes, increasing the scaffold N50 from 1.2 to 1.5 Gb and genome coverage from 30 to 42x. Herein, we have remapped and annotated the *A. mexicanum* Ig loci using the new genome version (UKY_AmexF1_1) and additional public transcriptomic data. As anticipated, the unusual orientation of the IGHJ-IGHC cluster regarding the IGHV gene cluster observed in the d/d strain was an assembly error. In addition, evidence of copy number variation (CNV) in the IGH and lambda (IGL) locus was found. The absence of the kappa locus and other observations regarding locus size withhold.

## 2 Results

### 2.1 *Ambystoma mexicanum* IGH locus

**Figure 1:**
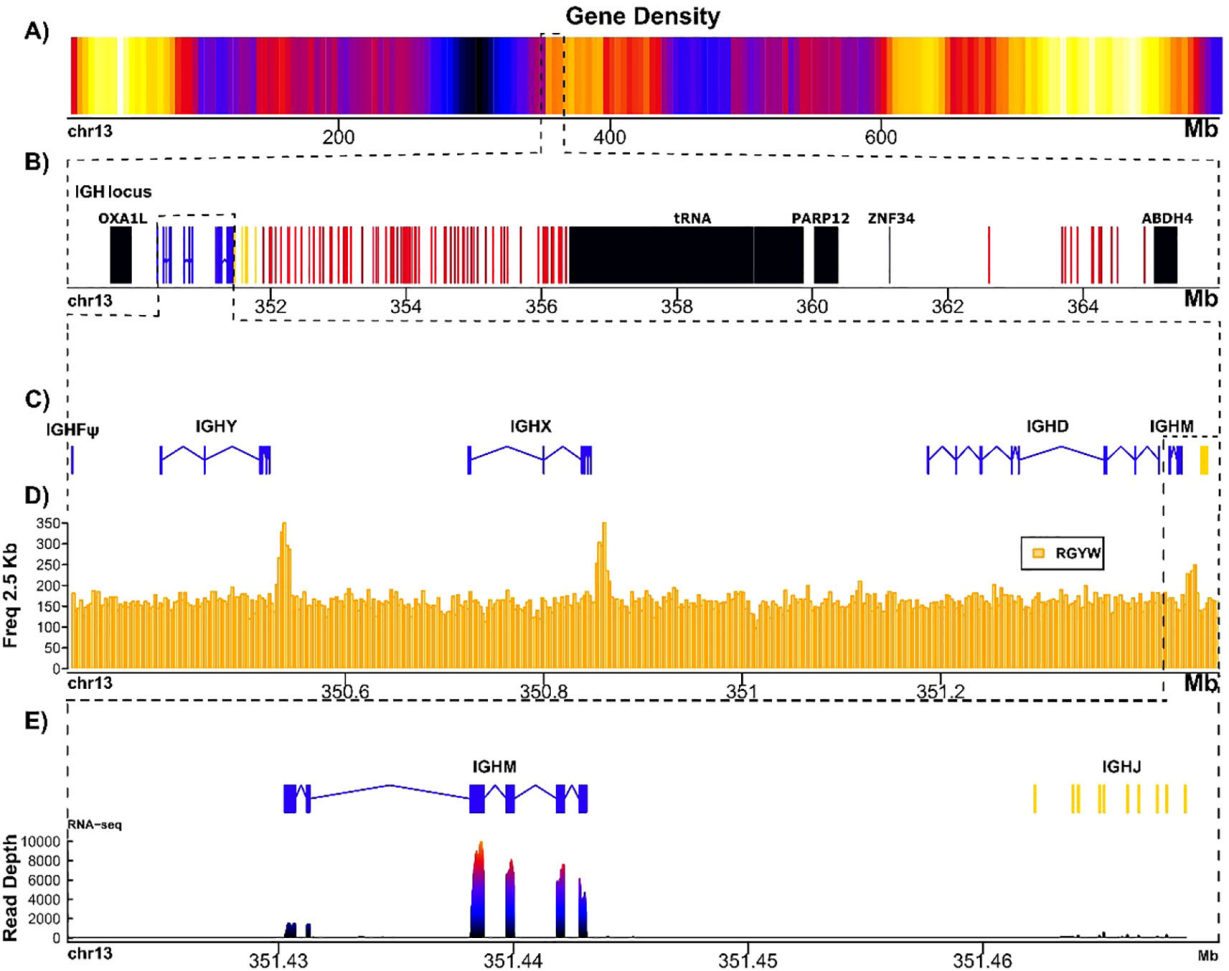
Schematic representation of heavy chain immunoglobulin loci in *A*. *mexicanum* genome (UKY). **(A)** The gene density in chr13. The color gradient from yellow to blue represents gene density, with yellow indicating high and blue low density. The black region corresponds to the centromere, which lacks genes. **(B)** Zoom of IGH locus is encoded, in black non-Ig genes, in blue the IGC genes, in yellow the IGHJ-D genes and in red the IGHV genes regardless if are functional or pseudogenes. The locus is in reverse direction; in the proximal flank the locus is flanked by OXA1L and ABDH4 in distal flank as we described in the V6 annotation. The IGHV cluster close to ABDH4 is separated to the rest of V genes by a tRNA´s cluster. **(C)** IGHM, IGHX and IGHY are encoded by four exons and its trans membranal and intra cytoplasmatic exons; IGHD encoded by 8 exons and its trans membranal and intra cytoplasmatic regions; IGHF was located in the locus as a pseudogene (ψ). **(D)** Frequency RGYW motif along to 2.5 kb in the IGHJ-C region, the peaks in frequency indicate the deamination hotspots targeted by AID to induce class switch recombination in IGHY, IGHX, and IGHM. **(E)** This panel shows the coverage of RNA-seq expression of the IGHM exons in spleen transcriptome.

The IGH locus maps at 350–364 Mbp in chromosome 13, next to the centromeric end of arm 13q, which is consistent with its localization in the d/d genome assembly (**Figure 1A**). Its size is also similar (14.5 Mbp) to what we previously described (12.1 Mbp). The syntenic relations are maintained between both genomes. The IGHV gene cluster is subdivided into two clusters, separated by a tRNA gene cluster, and a major improvement in the new assembly and annotation is that all IGHV genes are in the same transcriptional orientation as the IGHJ and IGHC gene cluster, confirming misassembly in AmbMex60DD (**Figure 1B**; **Supplementary Material 1****;****Supplementary Figure S1A**). The IGHV cluster is composed of 87 genes. Three additional IGHV pseudogenes, properly referred to as orphons (ORFons) because they are located outside the canonical loci (4), were found at the telomeric end of chr13q, and one was found in chr3 (644.5 Mbp) (**Supplementary Material 2****;****Supplementary Tables S1**, **S2**; GFF file).

Of the 87 IGHV genes within the IGH locus, 64 (73%) are functional. This result contrasts with our previous annotation in which only 47% of IGHV genes were reported as functional. As in the d/d strain, we found that 15% of functional IGHV genes had unusually long V-introns (> 150 pb). Also, as reported for the d/d strain, 64 IGHV functional genes belong to either clans II (42%) or III (54%), and two were indeterminate (clan I or III, 3.1%) (**Supplementary Material 2****;****Supplementary Tables S1**, **S2**).

No differences were observed regarding the number of IGHD, IGHJ and IGHC genes between AmbMex60DD and the current reference assembly. The IGHF gene is also a pseudogene. The switch regions (enriched in the RGYW motif) corresponding to IGHM, IGHX and IGHY were also identified in the expected positions (**Figures 1C, D**). The IGHM, IGHX and IGHY genes are actively transcribed in the spleen (**Figure 1E**).

In the comparative analysis of similarity-based correspondences among functional and IGHV pseudogenes, 67 BLAST best reciprocal pairs were identified between the AmbMex60DD and UKY_AmexF1_1 genome. In contrast, 20 IGHV genes from UKY_AmexF1_1 and 21 from AmbMex60DD lacked best match reciprocity (**Supplementary Material 1****;****Supplementary Figure S2**), suggesting IGHV allelic variation, CNV, or both (**Supplementary Material 3****;****Supplementary Tables S4**–**S6**).

### 2.2 *Ambystoma mexicanum* IGL locus

**Figure 2:**
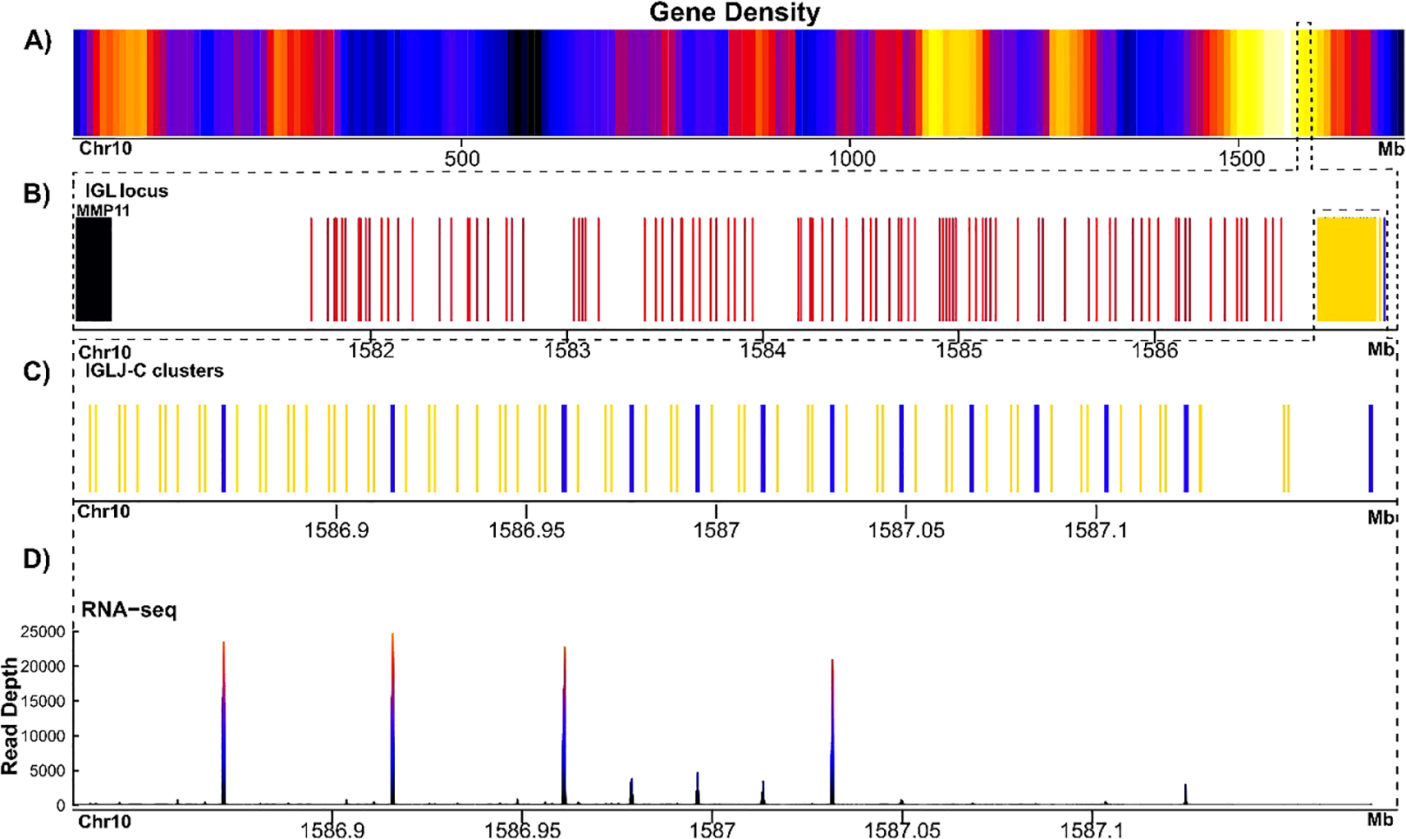
Schematic representation of immunoglobulin lambda locus in *A*. *mexicanum* genome (UKY) (on scale). **(A)** Gene density of chr10p. The color gradient from yellow to blue represents gene density, with yellow indicating high and blue low density. The black region corresponds to the centromere, which lacks genes. **(B)** Zoom of IGLV locus is encoded, in black non-Ig gene, in blue the IGLC genes, in yellow the IGLJ genes and in red the IGLV genes, regardless if they are functional or pseudogenes. The cluster IGLV is flanked by MMP11 gene in the proximal flank. **(C)** Close-up of IGLJ-C region, shows the 13 clusters, note that even the last pseudogenized cluster (ψ) is far away from the others J-C genes. **(D)** This panel shows the coverage of RNA-seq expression of the IGLC exons in spleen transcriptome.

The IGL locus maps at 1,581.6-1,587.1 Mbp in chr10p, spanning 5.5 Mbp (**Figures 2A, B**), and is shorter than our estimation for the IGL locus in AmbMex60DD assembly (9 Mbp). We observed substantial differences within the IGL locus. While our previous annotation (in AmbMex60DD) reported only three IGLJ-IGLC clusters, in UKY_AmexF1_1 we identified 13 IGLJ-IGLC clusters comprising 64 IGLJ genes (**Figure 2C****;****Supplementary Figure S1B****;****Supplementary Material 2**, **Supplementary Tables S1**, **S2****; GFF file**). We found a high degree of redundancy among amino acid sequences of IGLJ genes, with only 20 unique sequences. Most of the IGLJ genes are encoded by the identical sequences WMFGGGTQLNVL (18%), YVFGGGTQLSVL (17%), and YVLGGGTQLNVY (17%), while 11 sequences were unique (17%) (**Supplementary Material 1****;****Supplementary Figure S3A**).

Most of the IGLJ genes (96%) encoded the GGG tri-glycine motif instead of the canonical GXG di-glycine bulge. Consistent with our previous description, in 75% of sequences, the di- or tri-glycine motif is preceded by phenylalanine, and the remaining sequences are preceded by isoleucine or leucine (**Supplementary Material 1****;****Supplementary Figure S3B**). Almost the entire IGLJ–IGLC cluster was expressed in the spleen transcriptome. The IGLJ-IGLC 1–3 and 7 clusters displayed the highest expression levels, followed by IGLJ-IGLC 4–6 and 12 clusters. In contrast, the IGLJ–IGLC 8–10 and 11 clusters showed substantially lower expression. In the case of the most distal IGLJ-IGLC cluster, all IGLJ genes were pseudogenes. Consistently, as in the d/d strain, the most distal IGLC gene in UKY_AmexF1_1 (IGLC13) has no evidence of transcription based on spleen transcriptome analysis (**Figure 2D**).

In the remaining IGLC genes, we found evidence of active transcription, and high redundancy at the protein sequence level was observed, with trios (IGLC1-3, and IGLC5,7,12) being identical sequences. IGLC1 and IGLC2 from the d/d strain were identical to IGLC1–3 and IGLC5,7,12 counterparts in UKY_AmexF1_1, whereas IGLC3 in d/d was identical to IGLC13. In the comparative analysis with the other constant sigma, kappa, and lambda genes of *X. tropicalis* and *Homo sapiens*, the *A. mexicanum* IGLC genes grouped themselves, whether they were from UKY_AmexF1_1 or AmbMex60DD (**Supplementary Material 1****;****Supplementary Figures S4A, B**).

To address the degree of sequence similarity with IGLJ-IGLC clusters, including coding and non-coding intergenic sequence, we compared the nucleotide sequence of the 13 clusters against each other by calculating alignment coverage (AC) with an identity threshold of ≥ 95%. We observed that the first three clusters, which contain the largest number of IGLJ segments (10, 11), are more similar to each other than to the remaining 10 clusters. Clusters IGLJ-C4–11 display comparable sequence similarity among themselves, whereas IGLJ-C12 and IGLJ-C13 are less similar to all other clusters (**Supplementary Material 1****;****Supplementary Figure S5**).

The same analysis was conducted to compare the IGLJ-C clusters between both genomes. A high degree of similarity was observed between AmbMex60DD IGLJ-C1 and the first three IGLJ-C clusters of UKY, with 65.3% AC. AmbMex60DD IGLJ-C2 showed 91.2% AC with UKY_AmexF1_1 IGLJ-C12, and the AmbMex60DD J-C3 showed 79.8% AC with UKY_AmexF1_1 J-C13. Most of the remaining IGLJ-IGLC genes from the UKY_AmexF1_1 genome did not show substantial similarity to the three IGLJ-IGLC genes present in the AmbMex60DD genome, highlighting the divergence in the number and sequence conservation of IGLJ-IGLC genes among the two assemblies (**Supplementary Material 1****;****Supplementary Figure S6**).

In contrast with the 71 IGLV genes found in the AmbMex60DD genome, we found 94 IGLV genes in UKY_AmexF1_1, of which 79% are functional and 31% are pseudogenes (**Supplementary Material 2****;****Supplementary Tables S1**, **S2**). Reciprocal BLAST comparisons between IGLV genes found in both genomes revealed only 61 BLAST best reciprocal pairs, indicating a high degree of IGLV allelic variation and possibly copy number variation (**Supplementary Material 1****;****Supplementary Figure S7****) (****Supplementary Material 3****;****Supplementary Tables S7**–**S9**).

### 2.3 *Ambystoma mexicanum* IGS locus

**Figure 3:**
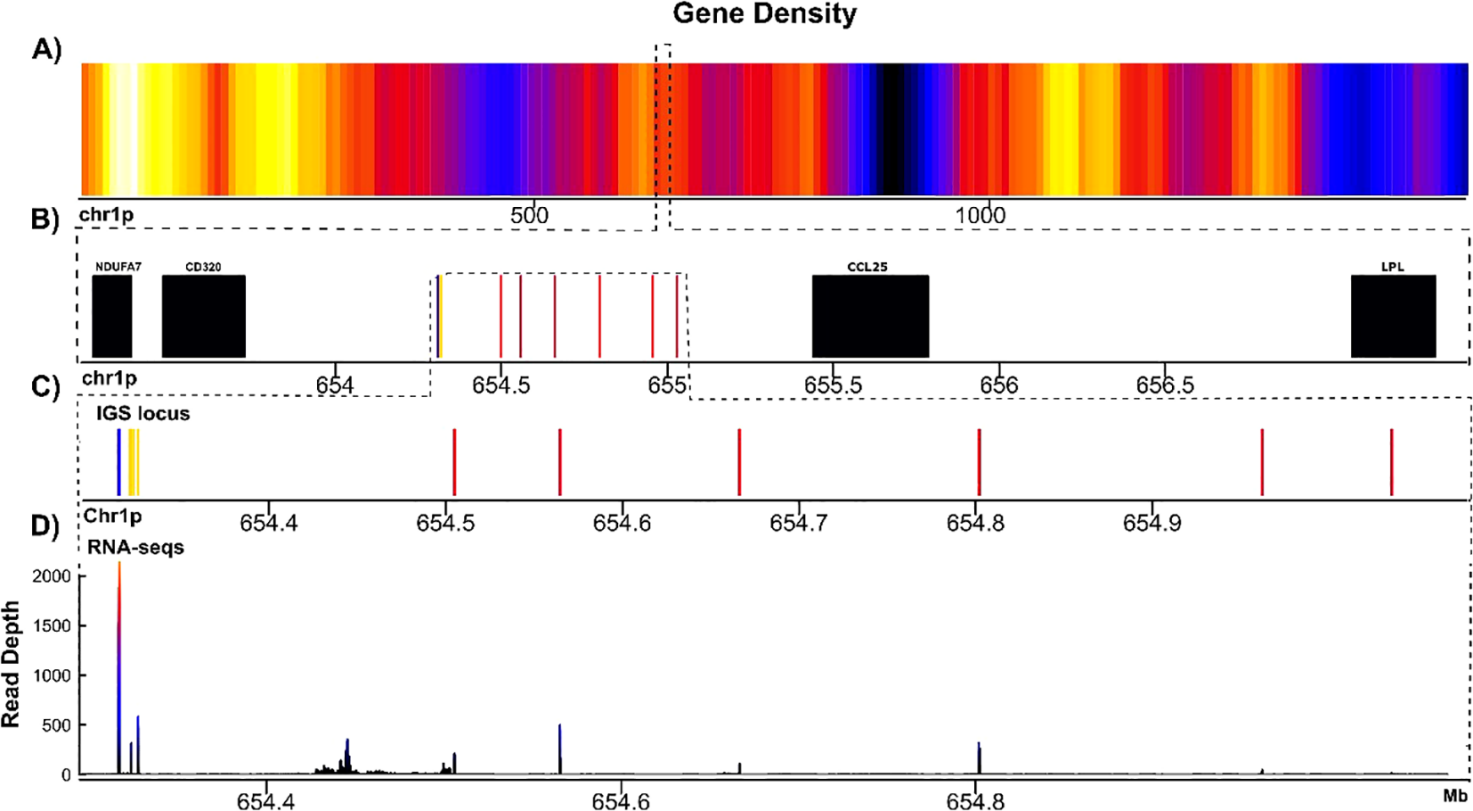
Schematic representation of the Immunoglobulin sigma locus in *A*. *mexicanum* genome (UKY) (on scale). **(A)** Gene density of chr1p. The color gradient from yellow to blue represents gene density, with yellow indicating high and blue low density. The black region corresponds to the centromere, which lacks genes. **(B)** Zoom of IGS locus is encoded, in black non-Ig genes, in blue the IGSC genes, in yellow the IGSJ genes and in red the IGSV genes, regardless if are functional or pseudogenes. The IGSV cluster is flanked by CD320 and NDUFA7 genes in the proximal flank and CCL25 and LPL in distal flank. **(C)** Close-up of only IGS-C-J-V genes. **(D)** This panel shows the coverage of RNA-seq expression of the IGSC-J-V exons in spleen transcriptome.

The sigma locus (IGS) maps to chr1p (654.3-655.0 Mbp), spanning approximately 0.73 Mbp (**Supplementary Material 2****;****Supplementary Tables S1**, **S2**; GFF file). As in the previous annotation, six functional IGSV genes were identified, a single IGSC gene, and four IGSJ genes. It is flanked by NUDFA7, CD320, CCL25, and LPL (**Figures 3A-C**) and has evidence of transcription on the spleen transcriptome (**Figure 3D**). In contrast with AmbMex60DD, all IGSV, IGSJ, and IGSC genes are in the same transcriptional orientation (**Supplementary Material 1****;****Supplementary Figure S1C**). As in AmbMex60DD, instead of the canonical FGXG motif encoding the di-glycine bulge observed in IGLJ and IGKJ genes, three of these IGSJ genes contain the FSXXS motif, and the IGSJ_001 exhibits an FGXXS motif.

### 2.4 Absence of the kappa chain in *Ambystoma mexicanum*

We previously reported the absence of the kappa locus in the *A. mexicanum* genome. Still, we identified the locus where it was expected to be, based on analysis of syntenic genes in *X. tropicalis* (chr1:2.1-8.0 Mbp), between the SUCLG1 and ADRA1D genes. Comparative analysis of the UKY_AmexF1_1 genome located this region in chr6p (1,486.6-1,522.4 Mbp), but no evidence of the kappa locus. It is worth noting that downstream of the SUCLG1 gene, there are two multi-exon genes (LOC138516801 and LOC138516802), one of them annotated as kappa immunoglobulin protein (XP_069488312.1). This gene contains six Ig domains, but with very weak similarity to IGKV (**Supplementary Material 1****;****Supplementary Figures S8A, B**). However, no evidence of RSS, IGKJ, and IGKC genes was identified nearby, indicating that if functional, such genes encode for non-kappa IgSF family proteins. Moreover, we did not find evidence of IGKV transcription in the *A. mexicanum* spleen transcriptome, confirming our previous description of the absence of the kappa locus in *A. mexicanum*.

## 3 Discussion

Genomic studies have revealed enormous plasticity in the organization of adaptive immunity receptor loci in vertebrates (5). Increasing the taxonomic spectrum is necessary for a detailed characterization of loci architecture and understanding the evolutionary forces shaping the germ-line adaptive immune receptor loci. Recent evidence suggests that human intra-species genomic variation is a common feature that represents an additional layer of complexity, contributing to the shaping of the adaptive immune receptor repertoire (6).

We have recently characterized the immunoglobulin loci in *A. mexicanum* of the d/d lab strain (1). Due to the great size of salamander genomes, the *A. mexicanum* genome assembly has been challenging and contains many gaps and unplaced scaffolds. We described some Ig gene orientations to be incompatible with VDJ recombination, which could be the result of the wrong orientation of some scaffolds (3). Here, we describe the Ig loci organization with a new genome assembly derived from the maternal pseudohaplotype of an F1 *A. mexicanum* x *A. tigrinum* F1 hybrid, which allowed us to clarify that indeed, some of the atypically oriented genes are technical artifacts. Moreover, this reanalysis confirms the loss of IGHF and the IGK locus described for the d/d strain and provides evidence of allelic single-nucleotide variation and copy number variation within *A. mexicanum* strains (3).

Regarding the IGH locus, we identified only two gaps and 87 IGHV genes, all of which are in the same orientation as the IGHJ-IGHC gene cluster, indicating that the AmbMex60DD genome contains wrongly oriented scaffolds. Differences in IGHV genes mapped to chr13 (87 in UKY_AmexF1_1 vs. 88 in AmbMex60DD) could represent a copy number variation (CNV). As for unmapped IGHV genes, we found 4 IGHV ORFons in UKY_AmexF1_1. When comparing the two genomes, we detected notable variability in the IGHV gene content between functional and pseudogenes (functional/pseudogene ratio of 2.7 in UKY_AmexF1_1 and 0.9 in AmbMex60DD). Further analysis is required to clarify if these differences may represent true CNV or if the pseudogene number in AmbMex60DD was overestimated due to a lower sequence coverage. It is worth noting that AmbMex60DD is an inbred strain, while the UKY_Amex_F1_1 assembly derives from a wild-type individual, reflecting the natural allelic diversity of the repertoire.

The observed IGHV sequence variation is consistent with evolutionary dynamics governed by birth-and-death processes (7), the accumulation of changes through genomic drift, and strain genetic diversity. The functional relevance of allelic variation has been demonstrated in human broadly neutralizing antibodies against influenza virus and SARS-CoV2 derived from the IGHV1–69 gene, where differences among alleles can alter the structural architecture and neutralizing capacity of the resulting antibodies (8, 9). Given that *A. mexicanum* is an endangered species, it is particularly relevant to consider factors that could compromise its survival. Among these, susceptibility to infections has been identified as a critical threat (10). In this context, the finding of CNV becomes significant, as it may influence the species’ immune responsiveness and, consequently, its vulnerability to pathogens (11, 12). Nevertheless, this connection should be viewed as a biological possibility rather than an established effect and will require experimental validation.

In tetrapods, IGHV genes are grouped into three clans (I, II, and III) that have persisted for approximately 370 million years (13). In *A. mexicanum* (UKY_AmexF1_1 genome), we identified 27 IGHV genes belonging to clans II and 35 to clan III; however, it was not possible to determine whether two additional genes belong to clan I or III, consistent with our previous report indicating the absence of clan I genes. The loss of clan I in *A. mexicanum* and the resulting evolutionary contraction of the IGHV repertoire could indicate additional mechanisms of primary antibody diversification (SHM and/or gene conversion) occurring in specialized anatomical sites, as suggested in birds, sheep, and rabbits (14).

Regarding the IGL locus, we observed a substantial difference in the number of IGLJ–C clusters between the UKY_AmexF1_1 genome and the previous AmbMex60DD annotation (12 functional IGLJ-C clusters in UKY_AmexF1_1 vs. 2 in AmbMex60DD). Because these genomes come from different individuals of the same species, this disparity could reflect an assembly artifact in UKY_AmexF1_1, loss of sequence information in the highly fragmented AmbMex60DD assembly, or genuine CNV between individuals. We found very little sequence diversity in IGLC coding sequences, suggesting that if this is a true CNV, the duplication event was very recent. We cannot rule out that such differences may be artefactual; however, the coverage-identity analysis reveals that, effectively, the differences observed in the number of IGLJ-C sets may result from true structural variation in the IGL locus, rather than misassembled haplotypes (15). Such variations are often mediated by non-allelic homologous recombination (15), which is facilitated by the presence of tandemly repeated gene segments. Evidence from other vertebrates supports this view. In humans, substantial structural diversity has been documented in the number of IGLJ–C3 cassettes among individuals, indicating that the IGLC region is highly variable in both copy number and allelic composition (16, 17). Similarly, comparative analyses in gorilla reveal an additional IGLJ2–IGLC2 tandem unit that shows almost complete sequence identity to the canonical copy, suggesting a very recent duplication event (18). Another pattern of structural dynamism in light chains of immunoglobulins occurs in lagomorphs, where the IGKC has also duplicated independently in different lineages, generating two copies with distinct structural features (19). Together, these findings suggest that the J–C regions of the immunoglobulin light chain loci are an evolutionarily dynamic region prone to recurrent rearrangements and lineage-specific expansions. Such structural differences could possibly influence the breadth and functional potential of the lambda light chain repertoire, including in axolotl. As reported in pre-genomic studies on the IGL in the axolotl, the majority of the repertoire’s light chain is dominated by the lambda chain (20). Here, we also observed a significant difference in the number of genes between the lambda and sigma chains, which may suggest a preferential usage of lambda chains. In the previous annotation, we reported for the first time the presence of the sigma chain in *A. mexicanum*, and we corroborate its presence in the UKY_AmexF1_1 genome with the cladistic marker FSXXS in the IGJS genes (3). Despite the evidence of sigma chain transcription, the repertoire of this chain is likely very limited in axolotl. The Igλ:Igσ ratio is highly variable between species (21) and requires further studies to elucidate if lambda and sigma-expressing cells represent functionally different B cell subsets.

The Igκ chain is expressed in almost all vertebrates, except birds and microchiropterans (21). However, despite its presence in some species, such as *X. tropicalis*, we confirm that the UKY_AmexF1_1 genome lacks the kappa locus, as previously described for the d/d lab strain. This observation was further strengthened by the lack of evidence of IGKV transcription in the *A. mexicanum* spleen transcriptome.

In conclusion, the availability of a high-quality genome enabled us to confirm and resolve several locus inconsistencies found in our previous characterization. Furthermore, in the case of the immunoglobulin loci, the UKY_AmexF1_1 genome reveals a structural organization that is more consistent with the underlying biology. In addition, we identified novel findings regarding CNV across the immunoglobulin loci in different axolotl strains. It remains unclear whether these differences result in immunophenotypic differences among individuals.

## 4 Methods

### 4.1 Ig loci mapping

We used the UKY_AmexF1_1 version of the *A. mexicanum* genome, released in May 2024 (https://www.ncbi.nlm.nih.gov/datasets/genome/GCF_040938575.1/). The genome was sequenced with the PacBio Sequel system and Illumina HiSeq technology from an adult F1 female progeny (isolate Mex_15411-BioSample SAMN41071122) of a cross between a female *A. mexicanum* and male *A. tigrinum* (isolate Tig_M23-BioSample SAMN43142724) (19), to build an F1 phased haploid assembly with HiFiasm, YAHS, and PreTextView v. JAN-2024, as an assembly method. To map V, J, and C genes, we used the corresponding *A. mexicanum* (cDNA and protein) sequences of the AmbMex60DD (DD151 isolate, white d/d genotype) version as input for homology searches using BLASTN, TBLASTX, and EXONERATE, yielding sequences with significant alignments BLAST e-value <1.0E-05 and >100 for Exonerate score as described (3) and were exported in GFF3 format. All gene models and sequence features were manually curated using the Integrative Genomics Viewer tool (IGV) (20).

Recombination signal sequences (RSSs), V, D, and J gene functionality were assigned, and a 42-bp region downstream of each putatively functional variable segment was aligned using ClustalX. The resulting alignments were manually curated with reference to the vertebrate consensus sequences of the heptamer (CACAGTG) and nonamer (ACAAAAAC).

Gene models were refined and validated by mapping transcriptome data (SRR5341570) from the spleen of *A. mexicanum* using the STAR aligner (21). Additional RNA-seq coverage big Wig (BW) data obtained from (spleen-SRR15610271; liver-SRR15610267; lung-SRR15610267, gills-SRR5042768, heart-SRR5042770), obtained from the NCBI FTP repository (https://ftp.ncbi.nlm.nih.gov/genomes/all/GCF/040/938/575/GCF_040938575.1_UKY_AmexF1_1/RNASeq_coverage_graphs/). An expressed gene was defined as a gene in which more than 10 RNA-seq reads mapped.

### 4.2 Assignment of VDJ functional status

The functionality of immunoglobulin heavy chain variable (IGHV) segments was evaluated using established IMGT criteria. For an IGHV segment to be classified as functional (F), it must meet several key requirements. First, it needs a predicted in-frame open reading frame (ORF) for both the signal peptide and the V-domain exon. Additionally, the presence of critical amino acids Cys23, Trp41, Trp52, and Cys104 within the V-domain exon. Finally, the segment must be followed by a canonical RSS as described above. The lack of the mentioned amino acids, signal peptide, expression in any transcriptome data, as well as the absence of a valid RSS, frame-shifted sequences, and the presence of stop codons were characteristics used to determine a non-functional or pseudogene. All numeric identifiers for Ig genes are provisional.

### 4.3 Class-switch region mapping

Raw counts were calculated within non-overlapping windows of 2,500 bp to identify the frequency of the 5´-RGYW-3´motif in the chr13 (350–364 Mbp) using the DNA-Pattern tool from RSA tools (22).

### 4.4 IGLJ-C lambda clusters analysis

Lambda light chain IGLJ–C clusters, comprising coding and non-coding regions, were aligned pairwise with Exonerate using a coverage identity cutoff of >95%.

### 4.5 Analysis of IGLC genes

Constant light chain gene sequences from *Homo sapiens*, *Mus musculus*, *Xenopus tropicalis*, and *A. mexicanum* were aligned using the PAM250 scoring matrix in Jalview v2.10.5 (23).

## Data Availability

The original contributions presented in the study are included in the article/**Supplementary Material**. Further inquiries can be directed to the corresponding authors.
