# Supplementary material for: An update on the characterization of immunoglobulin loci in Ambystoma mexicanum
Source: Front Immunol. 2026 Feb 23;17:1736245. doi: 10.3389/fimmu.2026.1736245 (PMC12967993; doi:10.3389/fimmu.2026.1736245)
Supplement: Supplementary file 1 [file DataSheet1.pdf]

Supplementary Material 1

- **Supplementary Figure S1.-** Comparative genomic organization of immunoglobulin light and heavy locus between the UKY_Amex_F1_1 and AmbMex60DD assemblies.

- **Supplementary Figure S2 .-** IGHV gene correspondence identified between the UKY_F1_1 and AmbMex60DD genomes assemblies of *Ambystoma mexicanum*

- **Supplementary Figure S3 .-** Frequency and percentage of IGLJ genes lambda genes.

- **Supplementary Figure S4.-** Analysis of IGL constant genes of *A. mexicanum* (UKY_F1_1 and AmbMex60DD), *X. tropicalis* and *H. sapiens*

- **Supplementary Figure S5 .-** Comparison of IGLJ-C lambda chain of the 13 clusters in UKY_F1_1 genome.

- **Supplementary Figure S6 .-** Comparison IGLJ-C lambda chain between the 13 clusters in UKY_F1_1 genome and the 3 IGLJ-C lambda chain clusters in AmbMex60DD genome.

- **Supplementary Figure S7 .-** IGLV gene correspondence identified between the UKY_F1_1 and AmbMex60DD genomes assemblies of *Ambystoma mexicanum*

- **Supplementary Figure S8.-** Synteny analysis of the abscense of kappa locus

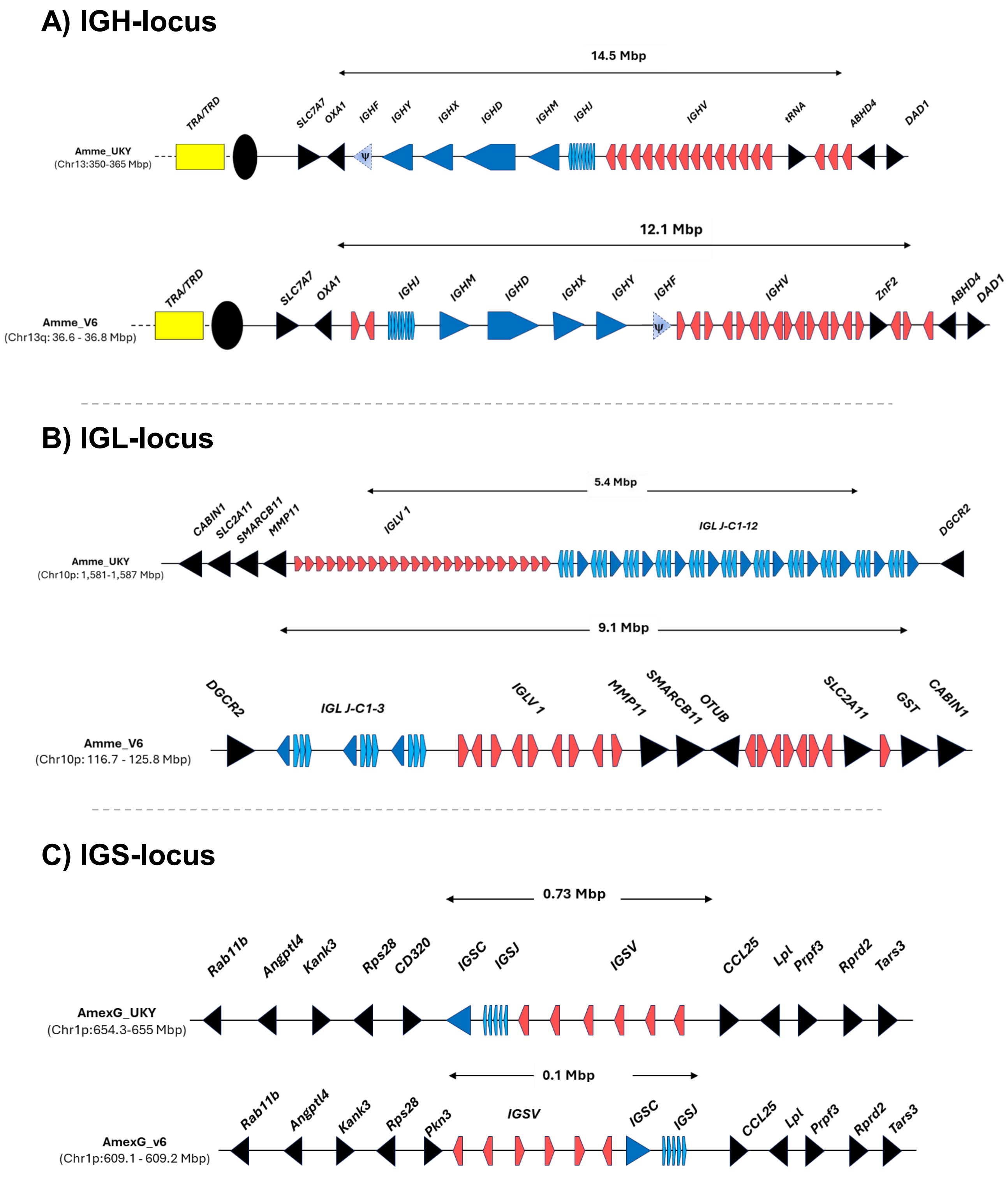

Supplementary Figure S1- Comparative genomic organization of immunoglobulin loci between the UKY_Amex_F1_1 and AmbMex60DD assemblies. V genes are depicted in red, C genes in dark blue, J genes in light blue, flanking non-Ig genes in black, and the TRA/TRD cluster in yellow. **A)** Gene orientation within the heavy chain (IGH) locus in the UKY_Amex_F1_1 and AmbMex60DD genomes. **B)** Gene orientation within the lambda chain (IGL) locus in the UKY_Amex_F1_1 and AmbMex60DD genomes. **C)** Gene orientation within the sigma chain (IGS) locus in the UKY_Amex_F1_1 and AmbMex60DD genomes. In all three panels, the gene orientation observed in the AmbMex60DD genome appears inconsistent with the expected configuration required for proper V(D)J recombination.

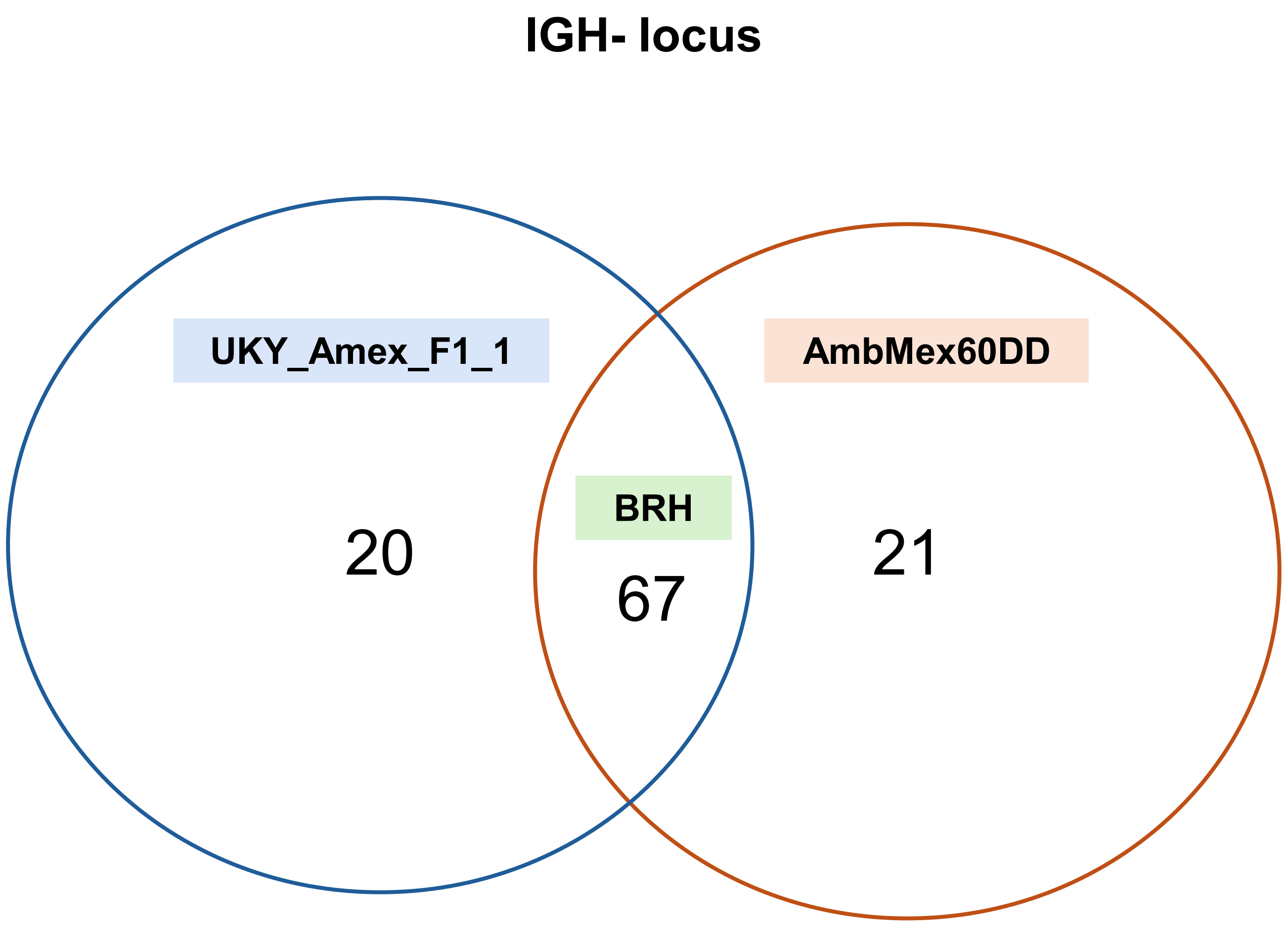

Supplementary Figure S2 .- IGHV BLAST best reciprocal hit (BRH). 67 IGHV best reciprocal pairs between AmbMex60DD and UKY_Amex_F1_1 genomes. 20 IGHV genes from UKY_Amex_F1_1 and 21 from AmbMex60DD did not have a reciprocal match.

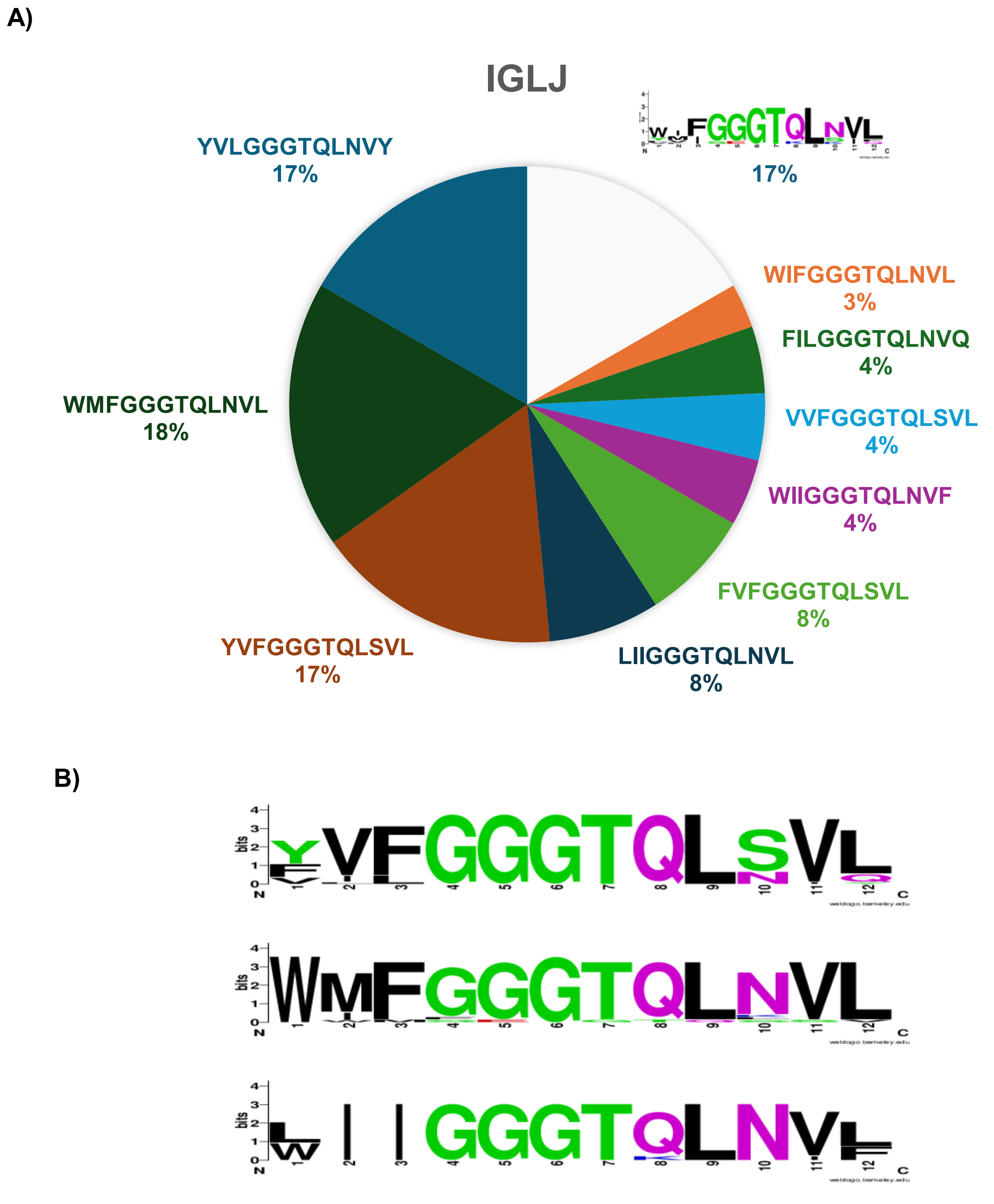

Supplementary Figure S3.- IGLJ genes of lambda *locus*. **A)** The percentage distribution of the 64 IGLJ segments that are repeated in the IGLJ-C 13 lambda clusters reported. Note the WMFGGGTQLNVL is the most repeated in the IGC clusters with the 18%, followed bye the YVFGGGTQLSVL and YVLGGGTQLNVY with 17%. The other 17% represented by a logo, are the 11 unique IGLJ sequences and, other sequences are presented at lower frecuencies ranging in 8% to 3%. **B)** The logos of all the IGLJ sequences represented as they grouped in a average distance phylogenetic análisis, the FGGG canocic motif is predominant in the first and second group, and in the third the Phenylalanine (F) is totally replaced by a Isoleucine (I).

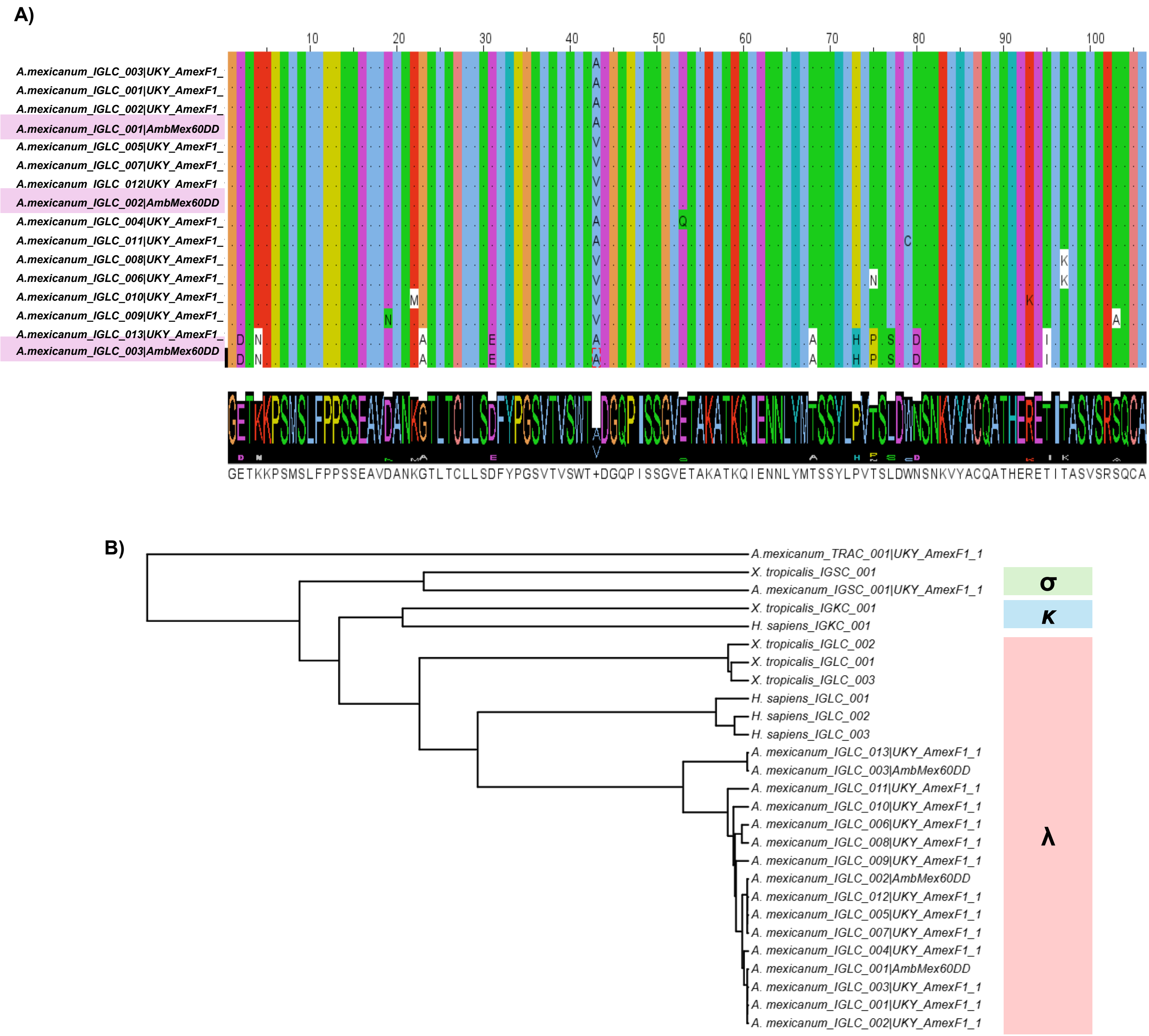

Supplementary Figure S4.- Analysis of constants genes of immunoglobulin ligth chains in *Homo sapiens*, *Xenopus tropicalis* and *Ambystoma mexicanum*. A) Aligment of the constant genes of the 13 IGLC UKY_AmexF1_1 and the 3 IGLC of AmbMex60DD, showing the no conserved aminoacids. The IGLC_001_AmbMex60DD is grouped with the IGLC1-3|UKY_AmexF1_1. The IGLC_002|AmbMex60DD is grouped with the IGLC_005, IGLC_007, IGLC_012|UKY_AmexF1_1. The IGLC_004, IGLC_011, IGLC_008 IGLC_006, IGLC_010, IGLC_009|UKY_AmexF1_1 presents a variation of one or two aminoacids between the others genes. The IGLC_003_ AmbMex60DD, IGLC_0013_ UKY_AmexF1_1.

B) The hierarchical structure shows the average distnace phylogenetic analysis. The C-lambda (λ) clade includes is clustered by specie, the C-sigma (σ) and C-kappa (κ) are in well-defined groups.

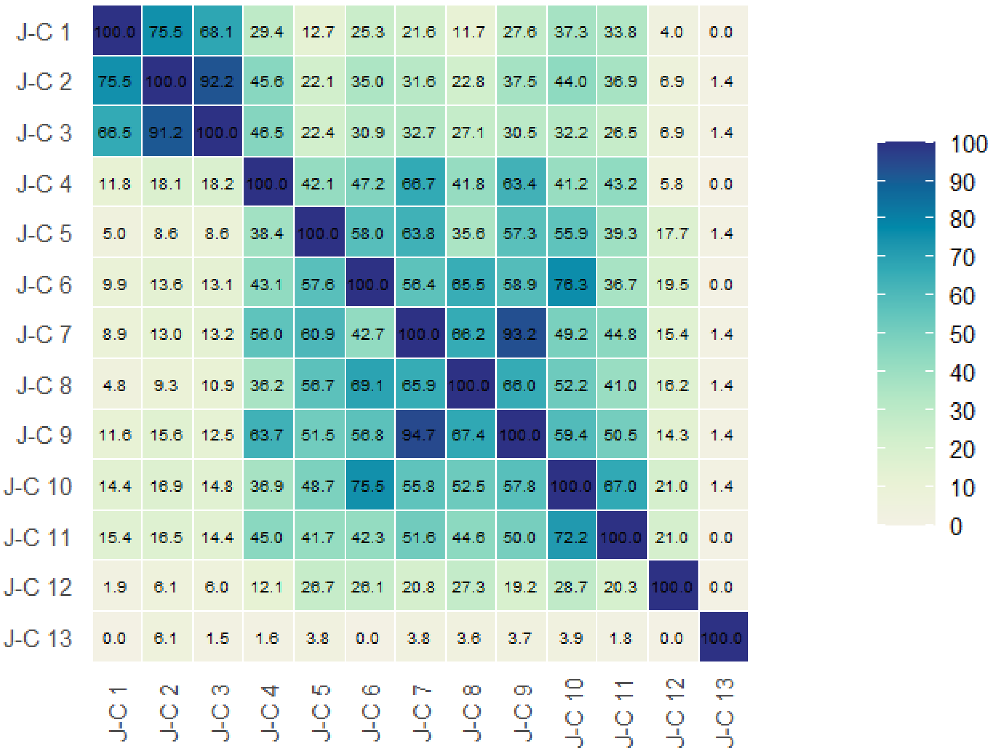

Supplementary Figure S5. Comparison of IGLJ-C clusters of UKY_AmexF1_1. Correlation of the percent sequence coverage above ≥95% identity of the 13 IGLJ-C clusters found in the UKY_AmexF1_1. There is high coverage between the IGLJ-C 1-3 clusters, whereas the J-C 4-11 clusters are similar to each other. Among all the functional clusters, cluster IGLJ-C12 exhibits the lowest coverage. The pseudogenized IGLJ-C 13 cluster has significantly less coverage than the other clusters.

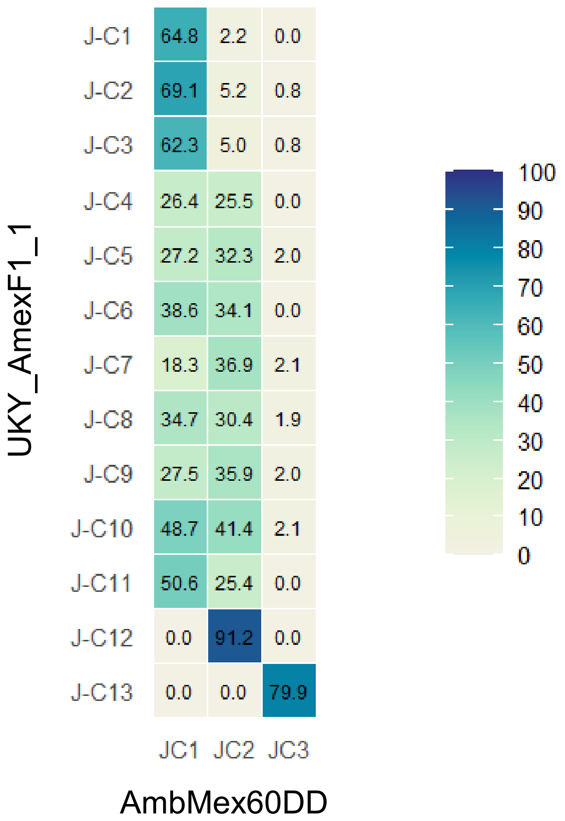

Supplementary Figure S6. Comparison of IGLJ-C clusters of UKY_AmexF1_1 and AmbMex60DD. Correlation of the percent sequence coverage above ≥95% identity of the 13 IGLJ-C clusters found in the UKY_AmexF1_1 versus the three IGLJ-C clusters previously found in the AmbMex60DD genome. The IGLJ-C1-V6 has the highest coverage with the IGLJ-C1-3-UKY clusters and with the IGLJ-C10-11-UKY clusters. The IGLJ-C2-V6 cluster has 91.24% coverage with the IGLJ-C-12-UKY cluster. The IGLJ-C-13-UKY and the IGLJ-C-3-V6 pseudogenized clusters presented the 79.9 % coverage aligment.

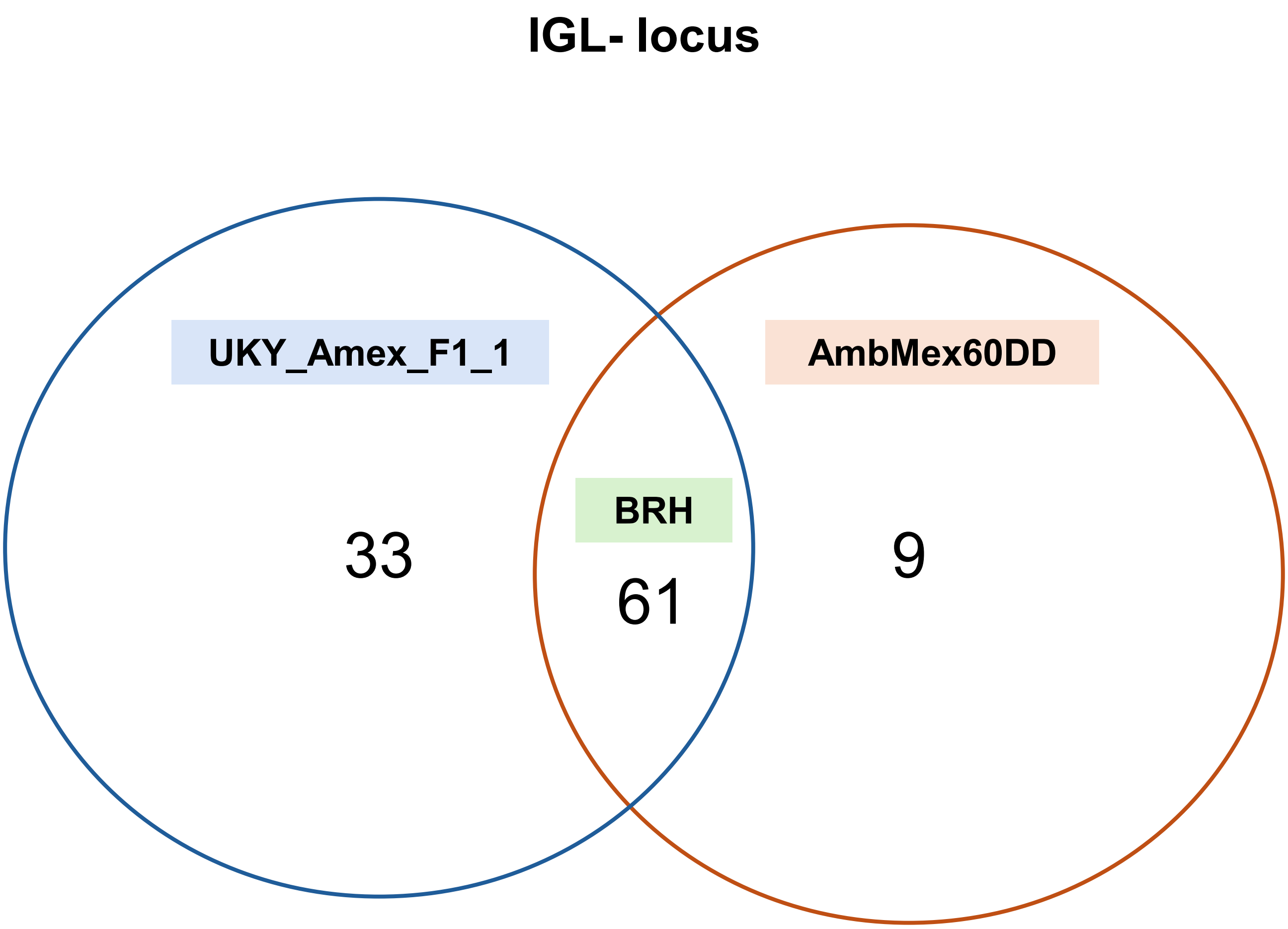

Supplementary Figure S7.- IGLV BLAST best reciprocal hit (BRH). 61 IGLV best reciprocal pairs between AmbMex60DD and UKY_Amex_F1_1 genomes. 33 IGLV genes from UKY_Amex_F1_1 and 9 from AmbMex60DD did not have a reciprocal match.

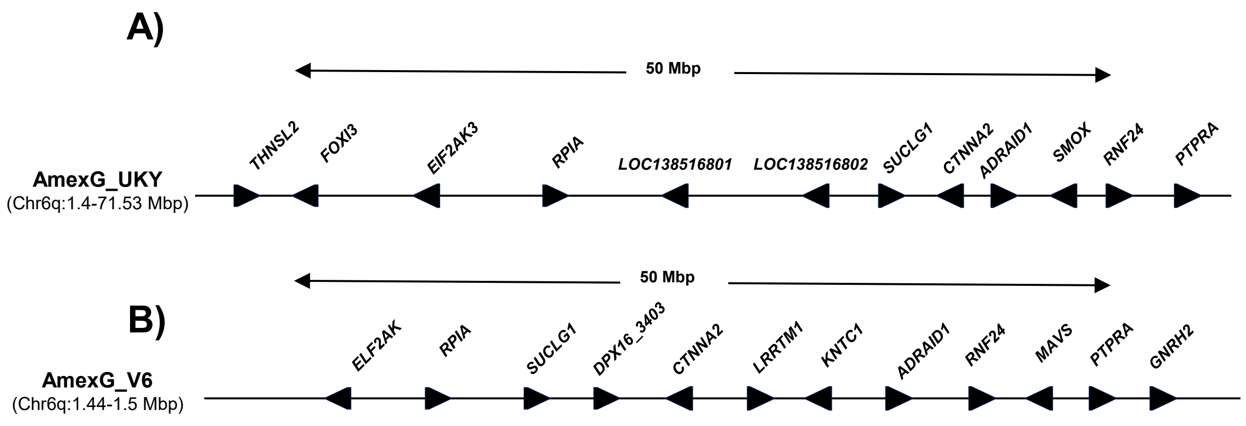

Supplementary Figure S8. Absence of the kappa chain gene in the UKY_AmexF1_1 genome. **A)** The BLAST analysis matched the annotation *LOC138516801* , whereas the adjacent annotation *LOC138516802* is identified as kappa; however, it shows low similarity despite containing six immunoglobulin-like exons. **B)** Compared to the V6 genome version, the position of several genes within this region has changed
